# Clinical benefit of sodium-glucose transport protein-2 inhibitors in patients with heart failure: An updated meta-analysis and trial sequential analysis

**DOI:** 10.3389/fcvm.2022.1067806

**Published:** 2022-12-02

**Authors:** Xiehui Chen, Lili Wang, Huijun Li, Weichao Huang, Siquan Huang, Lingyue Zhao, Wenqin Guo

**Affiliations:** ^1^Longhua District Central Hospital, Shenzhen, China; ^2^Fuwai Hospital Chinese Academy of Medical Sciences, Shenzhen, China; ^3^Shenzhen Longhua People’s Hospital, Shenzhen, China; ^4^Huazhong University of Science and Technology Union Shenzhen Hospital, Shenzhen, China

**Keywords:** sodium-glucose transport protein-2 inhibitors, heart failure, meta-analysis, trial sequential analysis, hospitalization due to HF

## Abstract

To assess whether the current body of accumulated data can give convincing evidence in favor of sodium-glucose transport protein-2 inhibitor (SGLT-2i) in all types of heart failure (HF). We searched for randomized controlled trials contrasting the effectiveness of SGLT-2i to placebo or other hypoglycemic medications on clinicaltrials.gov, PubMed, and the Cochrane Library database. To gauge effect size, hazard ratios (HR) were employed as measurements. The composite outcome of cardiovascular death or hospitalization owing to HF was the primary endpoint. Eleven studies were included. In comparison to the control group, the data demonstrated that SGLT-2i is related with a decreased incidence of composite outcome (HR: 0.77, 95% CIs: 0.73–0.81, *I*^2^ = 0%, *P* < 0.01), CV death (HR: 0.87, 95% CIs: 0.81–0.94, *I*^2^ = 3%, *P* < 0.01), all-cause mortality (HR: 0.90, 95% CIs: 0.84–0.96, *I*^2^ = 10%, *P* < 0.01), and hospitalization due to HF (HHF) (HR: 0.70, 95% CIs: 0.66–0.75, *I*^2^ = 0%, *P* < 0.01). The trial sequential analysis found strong evidence of a decrease in the incidence of all clinical outcomes with SGLT-2i when compared to the control group. Subgroup analysis demonstrated that the association between SGLT-2i and clinical outcome was independent of population characteristics. We confirm that the present evidence supports the use of SGLT-2i in a wide range of HF patients.

**Systematic review registration:**

[https://www.crd.york.ac.uk/prospero/#recordDetails], identifier [CRD42022333279].

## Introduction

Heart failure (HF) contributes to a global burden of disease. HF patients have a poor prognosis with an average 5-year survival rate of 46% (1). Sodium-glucose transport protein-2 inhibitors (SGLT-2i) are new hypoglycemic medications that prevent the kidney’s proximal convoluted tubules from reabsorbing glucose (2). Recently, evidence has shown that SGLT-2i deceased incidence of composite outcome of cardiovascular (CV) death or hospitalization due to HF (HHF) in patients with HF with reduced ejection fraction (HFrEF) (3, 4). Additional research has demonstrated the same advantages of SGLT-2i in HF patients with preserved EF (HFpEF) (5). Additionally, SGLT-2i reduced the composite outcome of CV mortality or HHF in patients with HF, regardless of the type of HF or the presence of diabetes, according to a pooled analysis of the EMPEROR-Preserved and EMPEROR-Reduced study (6). The 2022 American Heart Association/American College of Cardiology/Heart Failure Society of America (AHA/ACC/HFSA) guideline for the management of HF includes a class Ia recommendation for the use of SGLT-2i in patients with HFrEF; however, it includes a class IIa recommendation in patients with HFpEF, which suggests that the benefit of SGLT-2i in HFpEF is not entirely clear (7).

Recently, the results of the DELIVER trial, a large randomized study assessing efficacy of dapagliflozin in patients with HFpEF, were published at the European Society of Cardiology congress (8). This study was essential to draw a crucial conclusion about the advantages of SGLT-2i in all types of HF. Although studies have been performed to assess the therapeutic advantages of SGLT-2i in HF patients, these studies have limited power in evaluating the survival outcome. Accordingly, we conducted an updated meta-analysis to comprehensively review all the present evidence. Additionally, trial sequential analysis is analogous to an interim analysis in a clinical trial, where monitoring boundaries can be available to determine whether early termination of the trial is warranted when the *P*-value is small enough to show expected efficacy or ineffectiveness (9). Therefore, we also performed a trial sequential analysis to evaluate whether the current accumulated data could provide conclusive evidence in support of SGLT-2i in all types of HF.

## Methods

We reported the meta-analysis in accordance with the statement of Preferred Reporting Items for Systematic Reviews and Meta-Analyses. This study was registered with PROSPERO (CRD 42022333279).

### Search strategies and research sources

We searched for studies evaluating the effectiveness of SGLT-2i in PubMed, the Cochrane Library database, and clinicaltrials.gov. In order to find more related studies, we manually searched the bibliographies of the included studies and pertinent reviews. The keywords were: “SGLT-2i,” “empagliflozin,” “ertugliflozin,” “dapagliflozin,” “sotagliflozin,” “canagliflozin,” “licogliflozin,” “ipragliflozin,” “luseogliflozin,” and “heart failure.”

### Inclusion and exclusion criteria

Studies that satisfied the following criteria were included: (1) included patients with HF; (2) the intervention was SGLT-2i; (3) a placebo or other hypoglycemic medication served as the control group; (4) the outcome of interest was reported; and (5) randomized control trial (RCT). Studies that satisfied the following criteria were excluded: (1) included patients without HF or did not report the outcomes of HF subgroup; (2) the outcome of interest was not reported; (3) HR and its corresponding 95% confidence intervals (CIs) of the outcome of interest were not reported; and (4) non-RCT or a letter to the editor.

### Clinical outcome

The composite outcome of CV death or HHF was the primary endpoint. The all-cause mortality, CV death, and HHF were the secondary endpoints. The definition of all endpoints was consistent with the definition in each study.

### Data extraction and study quality assessment

The following information was independently retrieved by two researchers (WQ Guo and WC Huang) from the included studies: treatment regimen, comparator, population characteristics, mean follow-up time, and the outcomes. The quality of included studies was assessed using the Cochrane risk-of-bias tool. If the extracted information or quality assessment of the study was inconsistent between the two researchers, a third researcher made the final verdict.

### Statistical analysis

To gauge effect size, hazard ratios (HRs) were employed as measurements. HRs and corresponding 95% CIs were extracted as data from the included studies. The data was synthesized using the random effects model of the DerSimonian and Laird (10) method to account for unexplained heterogeneity. We used the Cochrane *Q*-test and the *I*^2^ index to assess heterogeneity between included studies (11). *I*^2^ values < 25, 25–50, 50–75, and >75% indicated no, mild, moderate, and high heterogeneity, respectively. Publication bias was assessed by drawing funnel plots (12). We performed subgroup analysis for all endpoints according to the EF value. Additionally, we performed a subgroup analysis to assess the relationship between population characteristics (such as gender, age, race, body mass index (BMI), New York Heart Association (NYHA) Class, estimated Glomerular Filtration Rate (eGFR), concomitant medication, atrial fibrillation status, diabetic status, and ischemia status) and composite outcomes.^14^ Meta-analyses were performed using R software version 4.0.3.

To test whether the meta-analysis is less rigorous than a good single randomized controlled trial, we performed a trial sequential analysis (TSA) to assess the reliability and conclusiveness of present evidence on the efficacy of SGLT-2 inhibitors. The relative risk reduction was calculated from the included studies; we set α = 5% and 1-β = 80%, and estimated sample size based on adjusted parameters. The trial sequential monitoring boundary was drawn for each study. When the boundary was not crossed, it indicated that more evidence might be needed to assess the efficacy of SGLT-2i; crossing the boundary indicated that the trial might be terminated early (9). The trial sequential analysis was conducted by TSA, version 0.9.5.10 Beta.

### Results

The flowchart of the study screening process is shown in Supplementary Figure 1. After searching the databases, 6,848 pieces of literature were screened by reviewing abstracts and titles. Finally, 11 studies were included in the analysis (3–5, 8, 13–19). Table 1 and Supplementary Table 1 illustrate the characteristics of the included studies and patients. Eleven studies report the composite outcome of CV death or HHF, 10 report the outcome of CV death, 9 report the outcome of all-cause death, and 10 report the outcome of HHF. One study included patients with HFpEF (5), one with HFmrEF and HFpEF (8), two with HFrEF (3, 4), three with both HFrEF and HFpEF (14–16), one with HFrEF, HEmrEF, and HFpEF (13), and three did not report the type of HF (17–19), respectively. Seven studies included patients with diabetes (13–19) and four with or without diabetes (3–5, 8). Median follow-up ranged from 9.2 to 50.4 months. Supplementary Figure 2 shows the quality assessment of the included studies. Overall, these studies were at low risk of bias.

**TABLE 1:** The characteristic of included studies.

Study	Year	Intervention	Control	The type of HF	The definition of HFpEF	The definition of HFrEF	Median follow-up (Months)	Outcomes*
SOLOIST-WHF	2020	Sotagliflozin	Placebo	HFpEF (19.5%); HFrEF (81.5%)	EF ≥ 50%	EF < 50%	9.2	1, 2, 3, 4
SCORE	2020	Sotagliflozin	Placebo	HFpEF (48.3%); HFmrEF (20.1%) HFrEF (31.6%)	EF ≥ 50%	EF < 40%	16	1, 2, 4
DECLARE-TIMI 58	2019	Dapagliflozin	Placebo	HFpEF (4.7%); HFrEF (3.9%)	EF ≥ 45%	EF < 45%	50.4	1, 2, 3, 4
VERTIS CV	2020	Ertugliflozin	Placebo	HFpEF (12.2%); HFrEF (5.8%)	EF ≥ 45%	EF < 45%	36	1, 2, 3, 4
EMPEROR-Preserved	2021	Empagliflozin	Placebo	HFpEF	EF ≥ 40%	–	26.2	1, 2, 3, 4
DAPA-HF	2019	Dapagliflozin	Placebo	HFrEF	–	EF ≤ 40%	18.2	1, 2, 3, 4
EMPEROR-Reduced	2020	Empagliflozin	Placebo	HFrEF	–	EF ≤ 40%	16	1, 2, 3, 4
CANVAS HF	2018	Canagliflozin	Placebo	NA	NA	NA	47	1, 2, 3, 4
CREDENCE	2019	Canagliflozin	Placebo	NA	NA	NA	31.4	1
EMPA-REG OUTCOME	2016	Empagliflozin	Placebo	NA	NA	NA	31.2	1, 2, 3, 4
DELIVER	2022	Dapagliflozin	Placebo	HFmrEF (33.8%) HHpEF (66.2%)	EF ≥ 50%	–	27.6	1, 2, 3, 4

HF, heart failure; EF, ejection fraction; HFrEF, heart failure with reduced ejection fraction; HFpEF, heart failure with preserved ejection fraction; NA, not available. *1, primary composite outcome; 2, cardiac death; 3, all-cause mortality; 4, hospitalization due to heart failure.

### Composite outcome of cardiovascular death or hospitalization due to heart failure

**FIGURE 1:**
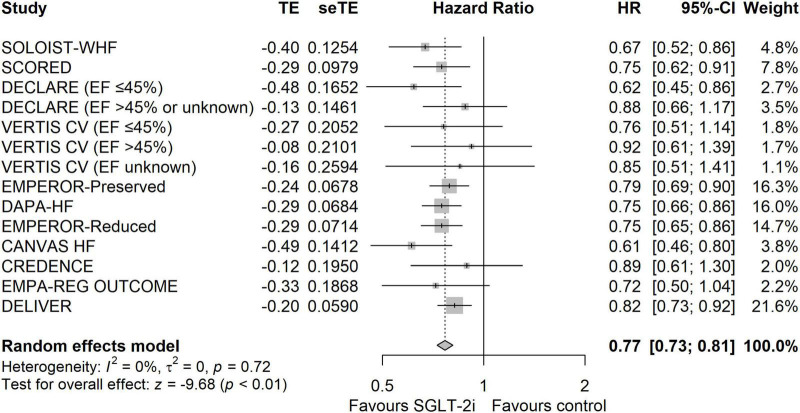
The forest plot of meta-analysis in terms of composite outcome of cardiovascular death or hospitalization due to heart failure.

The results showed that SGLT-2i was associated with a lower incidence of the composite outcome of CV death or HHF than the control group (HR: 0.77, 95% CIs: 0.73–0.81, *I*^2^ = 0%, *P* < 0.01) (Figure 1). The reduction of composite outcome was consistent among the patients with HFmrEF/HFpEF and HFrEF (HR: 0.78, 95% CIs: 0.73–0.85, *I*^2^ = 27 for HF with mildly reduced EF (HFmrEF)/HFpEF and HR: 0.75, 95% CIs: 0.69–0.82, *I*^2^ = 0 for HFrEF, Pinteraction = 0.45) (Supplementary Figure 6). Because the cut-off identifying the type of HF differed among studies, we performed a subgroup analysis according to the EF value. The results showed that the reduction in composite outcome was consistent for different EF value groups (Pinteraction = 0.77) (Supplementary Figure 7). The results of the subgroup analysis according to the population characteristics are shown in Supplementary Figures 11–21. The incidence of composite outcome was consistently reduced among the following groups: age <65 year (HR: 0.75, 95% CIs: 0.65–0.87, *I*^2^ = 0%), age ≥65 year (HR: 0.73, 95% CIs: 0.65–0.81, *I*^2^ = 0%), male (HR: 0.77, 95% CIs: 0.72–0.83, *I*^2^ = 1%), female (HR: 0.75, 95% CIs: 0.68–0.84, *I*^2^ = 0%), Asian (HR: 0.69, 95% CIs: 0.56–0.85, *I*^2^ = 45%), other (HR: 0.80, 95% CIs: 0.74–0.86, *I*^2^ = 4%), BMI < 30 kg/m^2^ (HR: 0.77, 95% CIs: 0.70–0.85, *I*^2^ = 10%), BMI ≥ 30 kg/m^2^ (HR: 0.76, 95% CIs: 0.69–0.84, *I*^2^ = 13%), eGFR < 60 ml/min/1.73 m^2^ (HR: 0.77, 95% CIs: 0.71–0.83, *I*^2^ = 15%), eGFR ≥ 60 ml/min/1.73 m^2^ (HR: 0.78, 95% CIs: 0.71–0.85, *I*^2^ = 0%), DM (HR: 0.76, 95% CIs: 0.70–0.83, *I*^2^ = 0%), Non-DM (HR: 0.78, 95% CIs: 0.71–0.85, *I*^2^ = 0%), AF (HR: 0.79, 95% CIs: 0.70–0.88, *I*^2^ = 0%), Non-AF (HR: 0.77, 95% CIs: 0.70–0.84, *I*^2^ = 0%), NYHA class II (HR: 0.72, 95% CIs: 0.65–0.79, *I*^2^ = 34%), NYHA class III–IV (HR: 0.85, 95% CIs: 0.77–0.94, *I*^2^ = 0%), ischemic (HR: 0.81, 95% CIs: 0.72–0.90, *I*^2^ = 0%), Non-ischemic (HR: 0.72, 95% CIs: 0.65–0.80, *I*^2^ = 0%), ARNI/ACEI/ARB (HR: 0.78, 95% CIs: 0.69–0.90, *I*^2^ = 13%), Non-ARNI/ACEI/ARB (HR: 0.72, 95% CIs: 0.63–0.83, *I*^2^ = 14%), MRA (HR: 0.76, 95% CIs: 0.69–0.83, *I*^2^ = 22%), and Non-MRA (HR: 0.74, 95% CIs: 0.66–0.84, *I*^2^ = 0%). The funnel plot showed no publication bias (Supplementary Figure 22).

**FIGURE 2:**
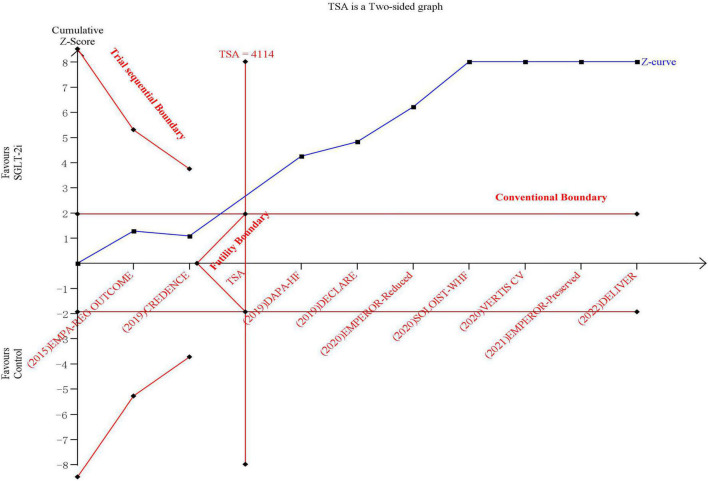
The trial sequential analysis in terms of composite outcome of cardiovascular death or hospitalization due to heart failure.

The results of TSA demonstrated that the cumulative *z*-curve crossed both the traditional boundary (*P* = 0.05) and line of required information size, indicating firm evidence for a reduction in the incidence of the composite outcome with SGLT-2i when compared with the control group (Figure 2).

### Cardiovascular death

**FIGURE 3:**
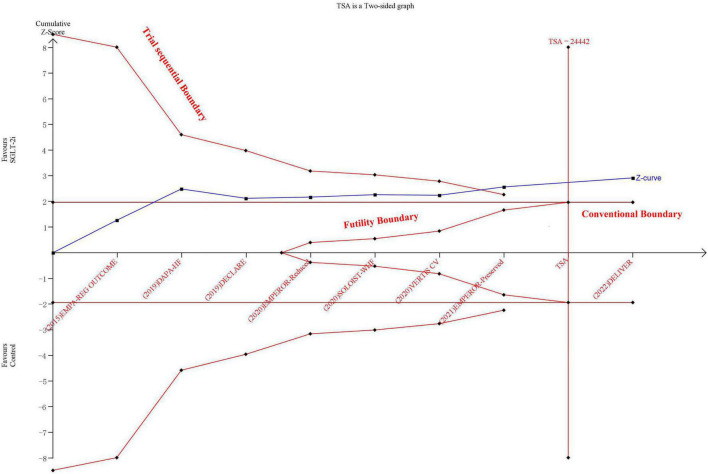
The trial sequential analysis in terms of cardiovascular death.

Sodium-glucose transport protein-2 inhibitor was associated with a lower risk of CV death than the control group (HR: 0.87, 95% CIs: 0.81–0.94, *I*^2^ = 3%, *P* < 0.01) (Supplementary Figure 3). The incidence of CV death was consistently reduced for HFrEF and HFmrEF/HFpEF groups (Pinteraction = 0.29) (Supplementary Figure 8). The results of TSA demonstrated that the cumulative *z*-curve crossed both the traditional boundary (*P* = 0.05) and trial sequential monitoring boundary, indicating firm evidence for a reduction in the incidence of CV death with SGLT-2i compared with the control group (Figure 3). The funnel plot showed no publication bias (Supplementary Figure 23).

### All-cause mortality

**FIGURE 4:**
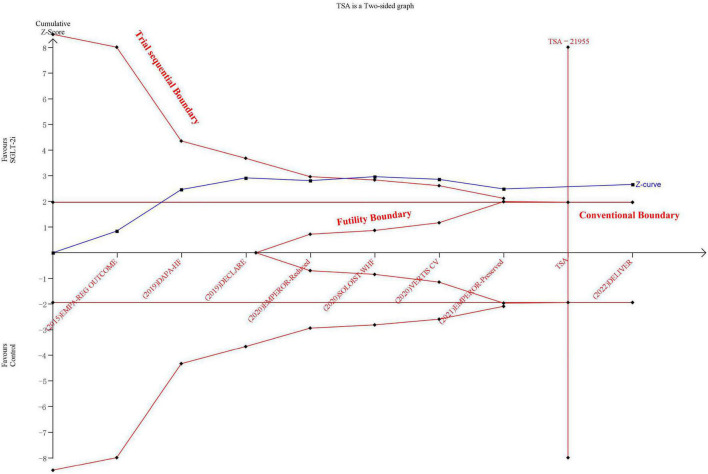
The trial sequential analysis in terms of all-cause mortality.

Sodium-glucose transport protein-2 inhibitor was associated with a lower incidence of all-cause mortality than the control group (HR: 0.90, 95% CIs: 0.84–0.96, *I*^2^ = 10%, *P* < 0.01) (Supplementary Figure 4). The incidence of all-cause morality was consistently reduced for HFrEF and HFmrEF/HFpEF groups (Pinteraction = 0.06) (Supplementary Figure 9). The results of TSA demonstrated that the cumulative *z*-curve crossed both the traditional boundary (*P* = 0.05) and trial sequential monitoring boundary, indicating firm evidence for a reduction in the incidence of all-cause mortality with SGLT-2i compared with the control group (Figure 4). The funnel plot showed no publication bias (Supplementary Figure 24).

### Hospitalization due to heart failure

Ten studies were included in terms of the HHF. The results showed that SGLT-2i was associated with a lower incidence of HHF than the control group (HR: 0.70, 95% CIs: 0.66–0.75, *I*^2^ = 0%, *P* < 0.01) (Supplementary Figure 5). The incidence of HHF was consistently reduced for HFrEF and HFmrEF/HFpEF groups (Pinteraction = 0.30) (Supplementary Figure 10). The results of TSA demonstrated that the cumulative *z*-curve crossed both the traditional boundary (*P* = 0.05) and line of required information size, indicating firm evidence for a reduction in the incidence of HHF with SGLT-2i when compared with the control group (Supplementary Figure 26). The funnel plot showed no publication bias (Supplementary Figure 25).

## Discussion

Our study had the following findings: SGLT-2i was associated with a lower incidence of the composite outcome, CV death, all-cause mortality, and HHF than the control group independent of the type of HF; the trial sequential analysis showed that the evidence confirmed the benefit of SGLT-2i inhibitors; the benefits of SGLT-2i are consistent across populations with different characteristics.

Previous studies demonstrated that SGLT-2i could decrease the incidence of cardiovascular events and HHF; beyond the hypoglycemic effect (20). Moreover, the cardiovascular protective effect of SGLT-2i treatment for HF patients was independent of diabetes status and type of HF (6, 14). Currently, the AHA/ACC/HFSA HF guidelines include a class Ia recommendation for the use of SGLT-2i in patients with HFrEF based on confirmed evidence; however, the guidelines include a class IIa recommendation for use of SGLT-2i in patients with HFpEF, which suggests that the benefit of SGLT-2i in HFpEF is not entirely clear (7). The DELIVER study, a second RCT evaluating efficacy of SGLT-2i in patients with HFpEF after the EMPEROR-Preserved trial, was important to update the evidence on the efficacy of SGLT-2i (8). These studies used the composite outcome of CV death or HHF as the primary endpoint, and the sample sizes were calculated to have sufficient statistical power to detect difference in the primary endpoint; however, they had limited ability to detect differences in survival endpoints such as CV death or all-cause mortality. In our study, we included the DELIVER study to perform an updated meta-analysis and the results suggested that the benefit of SGLT-2i in reducing the composite outcome was consistent between patients with HFrEF and HFpEF, with and without DM. In addition, the population benefiting from SGLT-2i was broad. Although increasing numbers of studies assessing the efficacy of SGLT-2i in patients with HF have been performed, analysis deciding whether more evidence was needed to support the use of SGLT-2i in HF patients was warranted. In our analysis, we performed a trial sequential analysis which confirmed that the evidence supports the use of SGLT-2i in patients with all types of HF; furthermore, our study confirmed that HF patients had the benefit of survival outcome from SGLT-2i.

Recently, many studies of the mechanism of the SGLT-2i effect in HF have been performed. At cellular level, heart failure commonly has fatty acid oxidation disorders, and impaired glucose uptake or oxidation, which can further cause myocardial dysfunction, while use of SGLT-2i can enhance hepatic synthesis, decrease urinary ketones, and cause mild or persistent hyperkeratosis (21). Ketone bodies synthesize ATP more efficiently than glucose or free fatty acids. Cardiovascular efficiency may be further improved when cardiometabolism shifts from fatty acid and glucose oxidation to ketone bodies, and the cardiovascular benefit associated with SGLT-2i therapy may be related to this altered energy metabolism (21). SGLT-2i can also participate in myocardial protection by improving myocardial ion homeostasis (22), autophagy (23), and changes in the regulation of adipokines (24). In addition, studies have revealed that SGLT-2i can improve the prognosis of patients with HF by inhibiting myocardial remodeling (25) and renal protection (26), reducing the risk of atrial fibrillation (27) and declining pulmonary artery pressure (28).

### Comparison with other studies

Previous meta-analyses have evaluated the efficacy of SGLT-2i. A study conducted by Cardoso et al. (29) including 15 trials and over 20,000 patients, found that SGLT-2i was associated with a lower incidence of all-cause mortality, CV death, and HHF in individuals with HF. More recently, two updated meta-analysis, including the latest DELIVER study, which showed that SGLT-2i significantly reduced the incidence of the composite outcome, CV death, all-cause mortality, and HHF in a broad range of patients with HF (30, 31). Our study differs from the above studies. We performed trial sequential analysis to confirm that the evidence supports the use of SGLT-2i; additionally, trial sequential analysis allows us to decide whether more evidence is needed, thereby avoiding redundant trials.

### Limitations

Our study has some limitations. Firstly, our study is based on the level of study; thus, we cannot assess all characteristics of the population and clinical outcomes. Secondly, the cut-off value identifying HFrEF, HFmrEF, and HFpEF differed among studies. However, we conducted a sensitivity analysis, including studies that defined EF less than 40% as HFrEF, EF between 40 and 50% as HFmrEF, and EF greater than 40% as HFpEF. The results showed that SGLT-2i was still associated with a lower composite outcome rate than the control group, independent of the type of HF. Thirdly, studies excluded because the HR and corresponding outcome of interest could not be extracted, may lead to selection bias; however, the sample size of these studies was too small to change the results of our study.

## Conclusion

It was confirmed that the present evidence supports the use of SGLT-2 inhibitors in a broad range of HF patients.

## Data availability statement

The raw data supporting the conclusions of this article will be made available by the authors, without undue reservation.

## Author contributions

WG and XC had full access to all the data in the study, took responsibility for the integrity of the data and the accuracy of the data analysis, designed the research, and wrote and revised the manuscript. XC, LZ, HJL, and LW directed the revision of the manuscript. WH and WG performed data searches and conducted data selection. WG, SH, and LZ helped with data analysis. All authors contributed to the article and approved the submitted version.

## Abbreviations

SGLT-2i: sodium-glucose transport protein-2 inhibitor
HR: hazard ratio
HF: heart failure
CV: cardiovascular
HFrEF: HF with reduced ejection fraction
HFpEF: HF with preserved EF
HHF: hospitalization due to HF
CIs: confidence intervals
TSA: trial sequential analysis
HFmrEF: HF with mildly reduced EF.
