# Supplementary material for: Clinical benefit of sodium-glucose transport protein-2 inhibitors in patients with heart failure: An updated meta-analysis and trial sequential analysis
Source: Front Cardiovasc Med. 2022 Dec 2;9:1067806. doi: 10.3389/fcvm.2022.1067806 (PMC9755323; doi:10.3389/fcvm.2022.1067806)

Supplemental File

Supplemental Table 1. The characteristic of included patients.
Supplemental Figure 1. The flowchart of the study screening process.
Supplemental Figure 2. The quality assessment of included studies.
Supplemental Figure 3. The forest plot of meta-analysis in terms of cardiovascular death.
Supplemental Figure 4. The forest plot of meta-analysis in terms of all-cause mortality.
Supplemental Figure 5. The forest plot of meta-analysis in terms of hospitalization due to heart failure.
Supplemental Figure 6. The subgroup analysis of primary outcome according to the type of heart failure.
Supplemental Figure 7. The subgroup analysis of primary outcome according to the ejection fraction (EF) value.
Supplemental Figure 8. The subgroup analysis of cardiovascular death according to the type of heart failure.
Supplemental Figure 9. The subgroup analysis of all-cause mortality according to the type of heart failure.
Supplemental Figure 10. The subgroup analysis of hospitalization due to heart failure according to the type of heart failure.
Supplemental Figure 11. The subgroup analysis of primary outcome according to the age.
Supplemental Figure 12. The subgroup analysis of primary outcome according to the sex.
Supplemental Figure 13. The subgroup analysis of primary outcome according to the race.
Supplemental Figure 14. The subgroup analysis of primary outcome according to the body mass index (BMI).
Supplemental Figure 15. The subgroup analysis of primary outcome according to the estimated glomerular filtration rate (eGFR).
Supplemental Figure 16. The subgroup analysis of primary outcome according to the diabetes mellitus (DM).
Supplemental Figure 17. The subgroup analysis of primary outcome according to the atrial fibrillation/flutter (AF).
Supplemental Figure 18. The subgroup analysis of primary outcome according to the NYHA Class.
Supplemental Figure 19. The subgroup analysis of primary outcome according to the heart failure etiology.
Supplemental Figure 20. The subgroup analysis of primary outcome according to the use of ARNI (angiotensin receptor-neprilysin inhibitor)/angiotensin converting enzyme inhibitor (ACEI)/ angiotensin receptor blocker (ARB).
Supplemental Figure 21. The subgroup analysis of primary outcome according to the use of mineralocorticoid receptor antagonists (MRAs).
Supplemental Figure 22. The funnel plot in terms of primary outcome.

Supplemental Figure 23. The funnel plot in terms of cardiovascular death.
Supplemental Figure 24. The funnel plot in terms of all-cause mortality.
Supplemental Figure 25. The funnel plot in terms of hospitalization due to heart failure.
Supplemental Figure 26. The trial sequential analysis in terms of hospitalization due to heart failure.

Supplemental Table 1. The characteristic of included patients.

Study	Year	Intervention	Control	Age (Years)	Male (%)	BMI (kg/m^2)	Median NT-proBNP (pg/ml)	Hypertension (%)	DM (%)	Ischemic HF (%)	AF (%)
SOLOIST-WHF	2020	Sotagliflozin	Placebo	70.0	66.3	30.8	1779	NA	100.0	NA	NA
SCORE	2020	Sotagliflozin	Placebo	69.0	55.1	31.8	197	NA	100.0	NA	NA
DECLARE-TIMI 58	2019	Dapagliflozin	Placebo	63.9	62.6	32.0	NA	92.9	100.0	54.3	NA
VERTIS CV	2020	Ertugliflozin	Placebo	64.5	68.1	32.6	NA	93.4	100.0	NA	NA
EMPEROR-Preserved	2021	Empagliflozin	Placebo	71.8	55.4	29.8	970	90.6	49.1	35.4	51.1
DAPA-HF	2019	Dapagliflozin	Placebo	66.4	76.6	28.1	1437	NA	41.8	56.4	38.3
EMPEROR-Reduced	2020	Empagliflozin	Placebo	67.3	76.1	27.9	1906	72.3	49.80	51.7	36.7
CANVAS HF	2018	Canagliflozin	Placebo	63.8	55.6	33.2	NA	95.3	100	NA	14.4
CREDENCE	2019	Canagliflozin	Placebo	63.0	66.1	31.3	NA	96.8	100	NA	NA
EMPA-REG OUTCOME	2016	Empagliflozin	Placebo	63.1	71.2	30.6	NA	NA	100	NA	NA
DELIVER	2022	Dapagliflozin	Placebo	71.6	56.1	NA	NA	88.7	55.2	NA	42.2

HF=heart failure; EF=ejection fraction; HFrEF=heart failure with reduced ejection fraction; HFpEF=heart failure with preserved ejection fraction; BMI=body mass index; DM=diabetes mellitus; AF=atrial fibrillation; NA=not available.

Supplemental Figure 1. The flowchart of the study screening process.

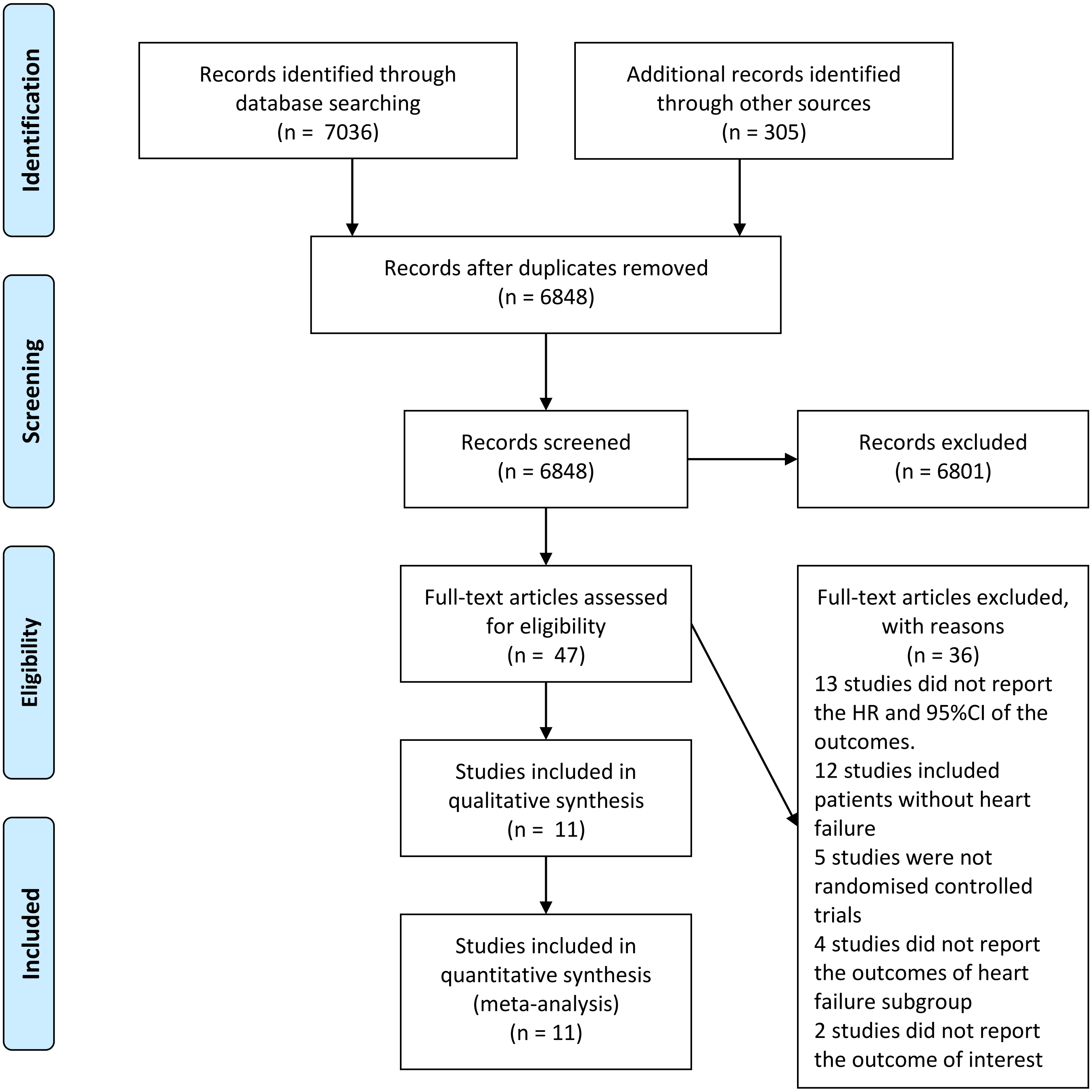

Supplemental Figure 2. The quality assessment of included studies.

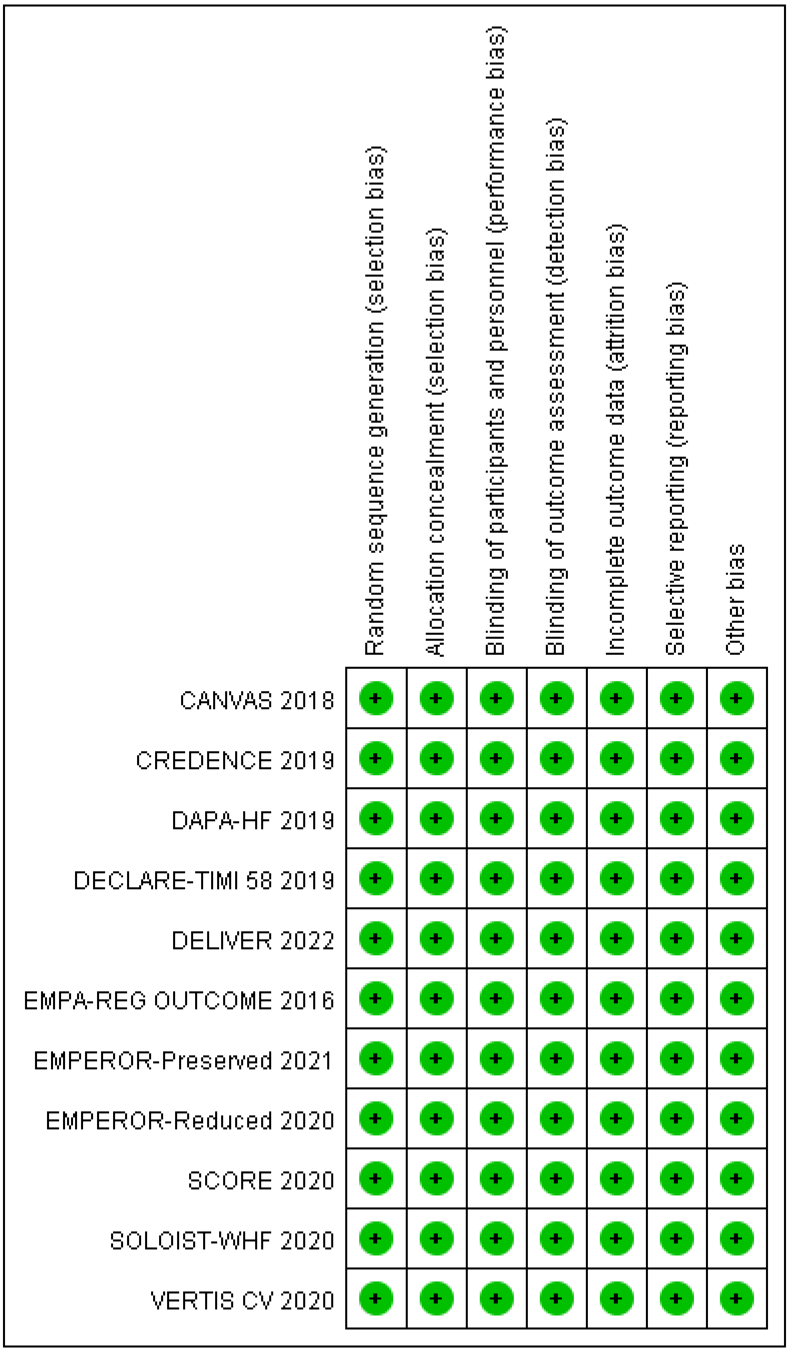

Supplemental Figure 3. The forest plot of meta-analysis in terms of cardiovascular death.

Study	TE	seTE	HR	95%-CI	Weight
SOLOIST-WHF	-0.17	0.1897	0.84	[0.58; 1.22]	4.2%
SCORED	-0.15	0.1455	0.86	[0.65; 1.14]	7.1%
DECLARE (EF ≤45%)	-0.60	0.2483	0.55	[0.34; 0.89]	2.4%
DECLARE (EF >45% or unknown)	0.34	0.2114	1.41	[0.93; 2.13]	3.4%
VERTIS CV (EF ≤45%)	-0.04	0.2665	0.96	[0.57; 1.62]	2.1%
VERTIS CV (EF >45%)	0.08	0.2638	1.08	[0.64; 1.81]	2.2%
VERTIS CV (EF unknown)	-0.22	0.2857	0.80	[0.46; 1.40]	1.8%
EMPEROR-Preserved	-0.09	0.0920	0.91	[0.76; 1.09]	17.7%
DAPA-HF	-0.20	0.0895	0.82	[0.69; 0.98]	18.7%
EMPEROR-Reduced	-0.08	0.1023	0.92	[0.75; 1.12]	14.3%
CANVAS HF	-0.33	0.1768	0.72	[0.51; 1.02]	4.8%
EMPA-REG OUTCOME	-0.34	0.2532	0.71	[0.43; 1.17]	2.3%
DELIVER	-0.13	0.0893	0.88	[0.74; 1.05]	18.8%
**Random effects model**			**0.87**	**[0.81; 0.94]**	**100.0%**

Heterogeneity: $I^2 = 3\%$, $\tau^2 < 0.0001$, $p = 0.42$
Test for overall effect: $z = -3.56$ ($p < 0.01$)

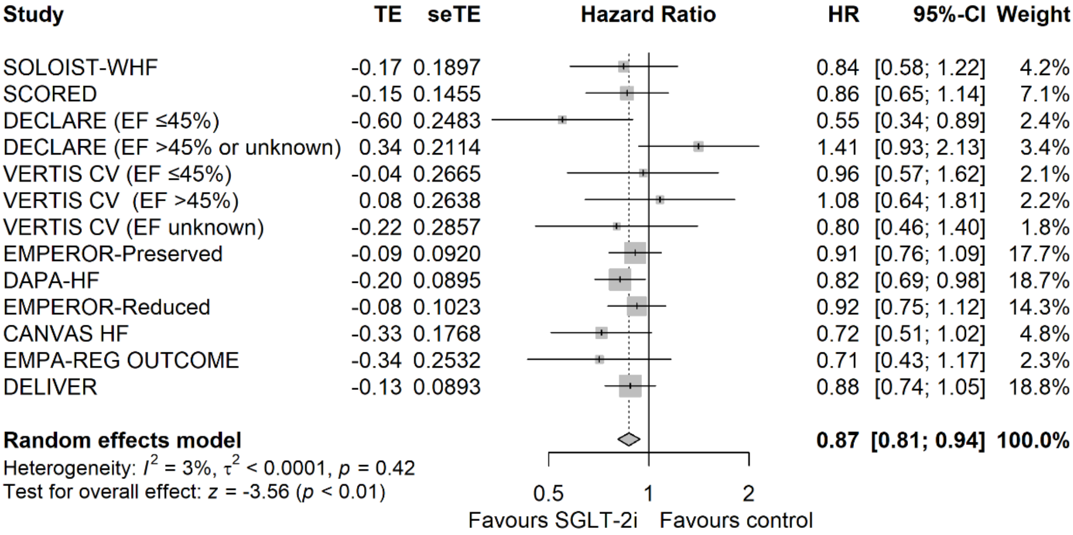

Supplemental Figure 4. The forest plot of meta-analysis in terms of all-cause mortality.

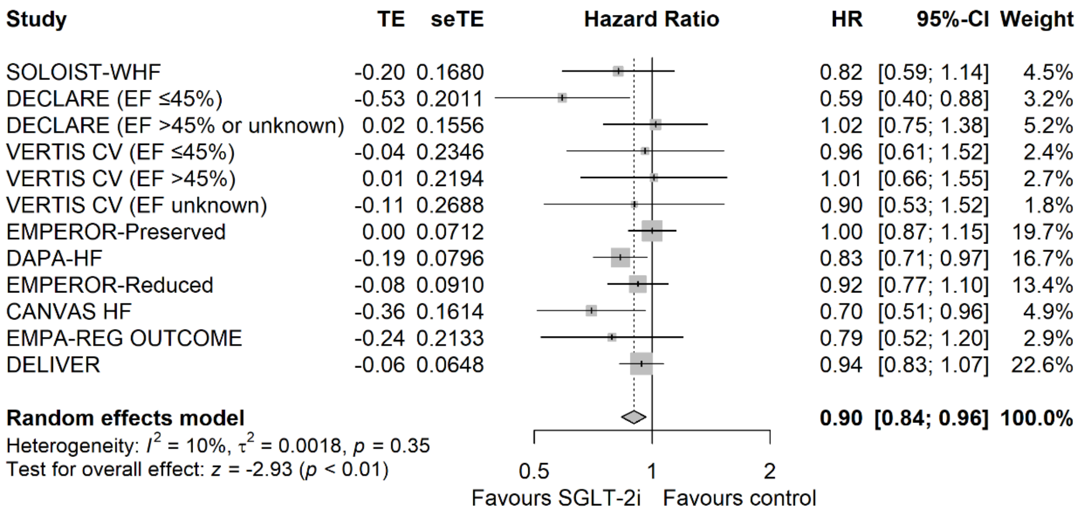

Supplemental Figure 5. The forest plot of meta-analysis in terms of hospitalization due to heart failure.

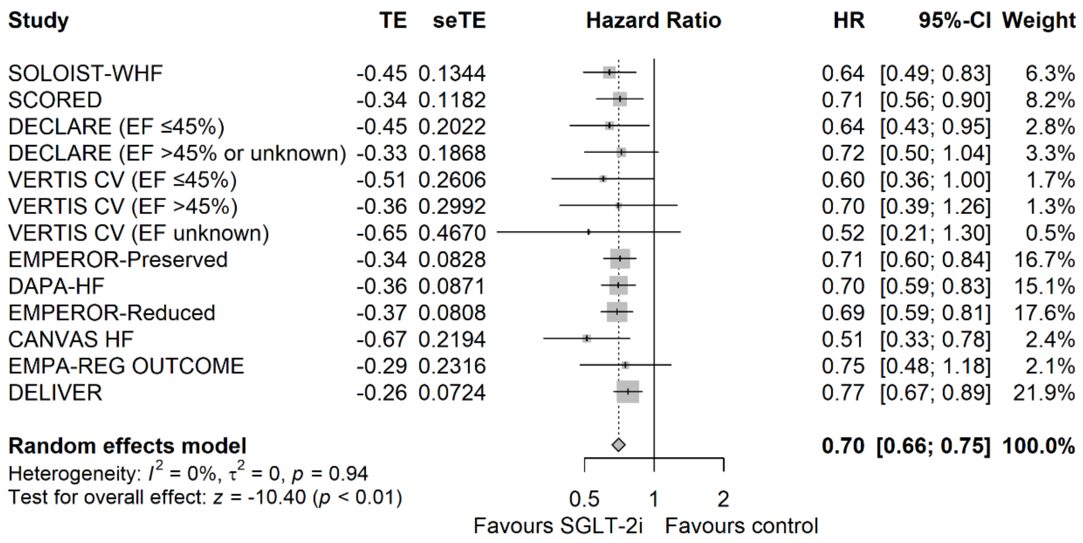

Supplemental Figure 6. The subgroup analysis of primary outcome according to the type of heart failure.

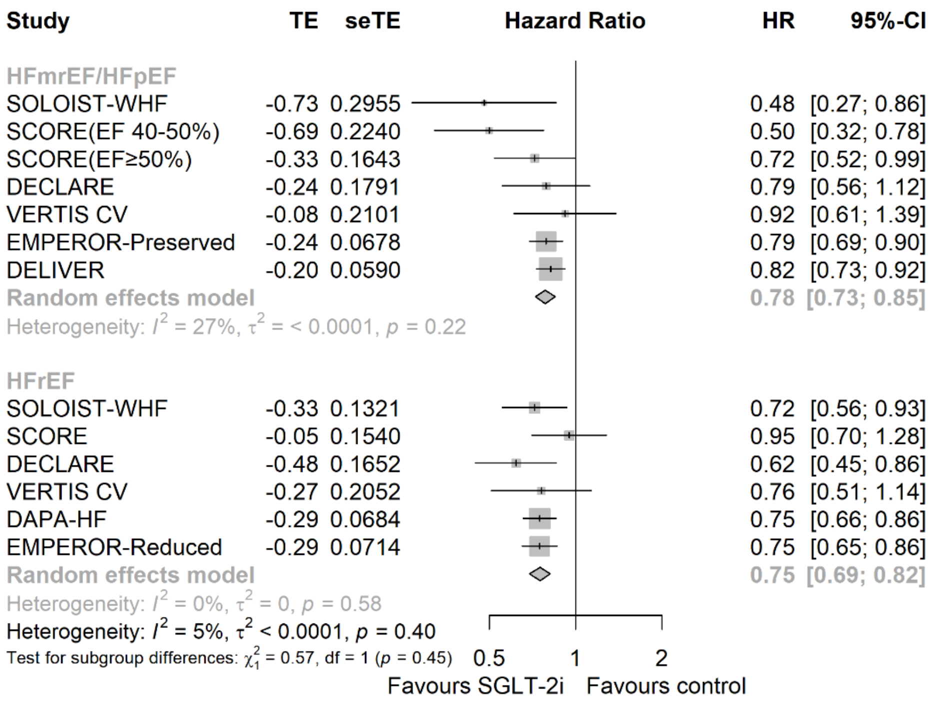

Supplemental Figure 7. The subgroup analysis of primary outcome according to the ejection fraction (EF) value.

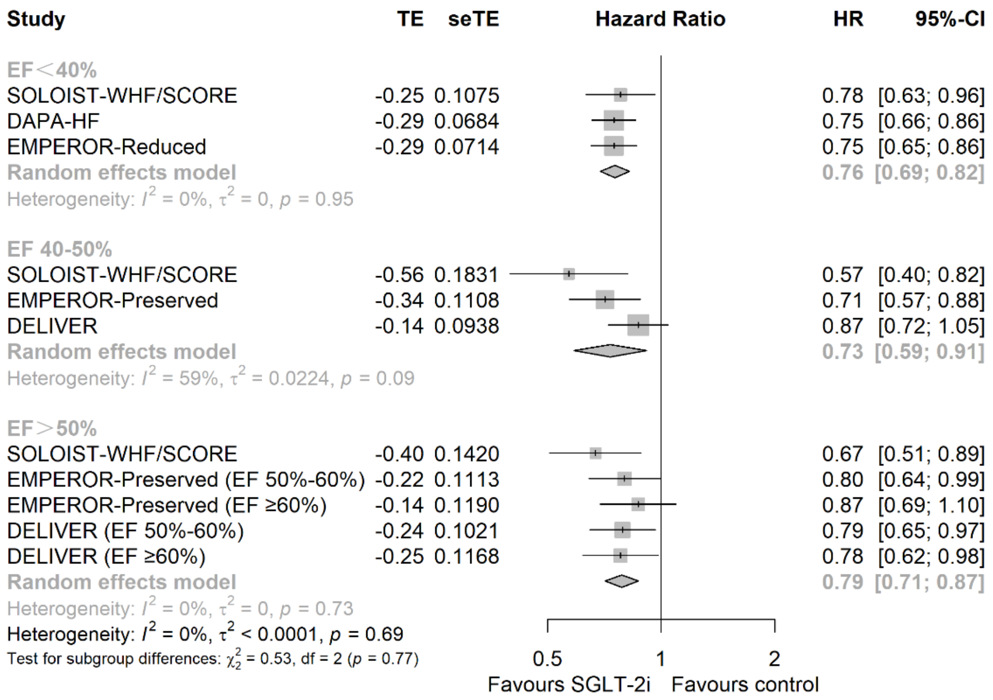

Supplemental Figure 8. The subgroup analysis of cardiovascular death according to the type of heart failure.

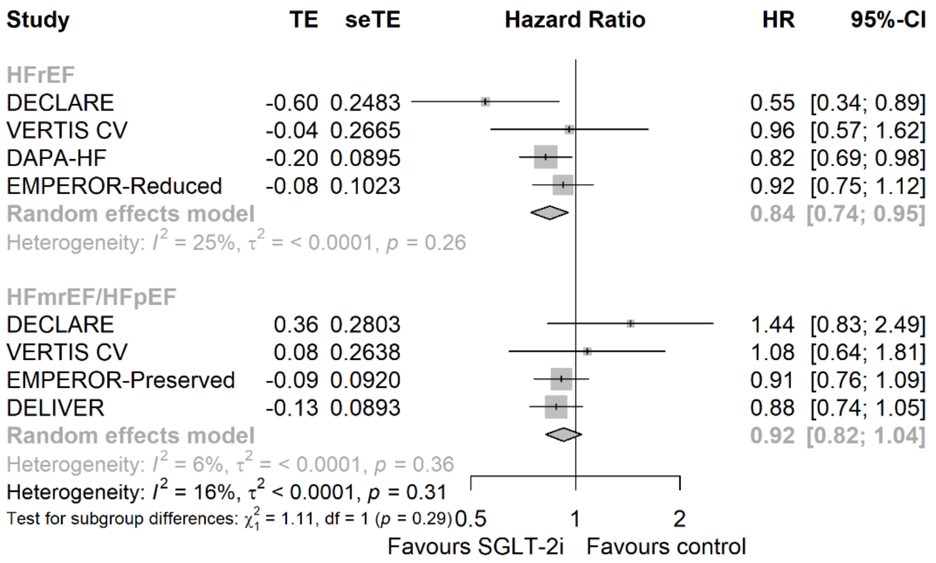

Supplemental Figure 9. The subgroup analysis of all-cause mortality according to the type of heart failure.

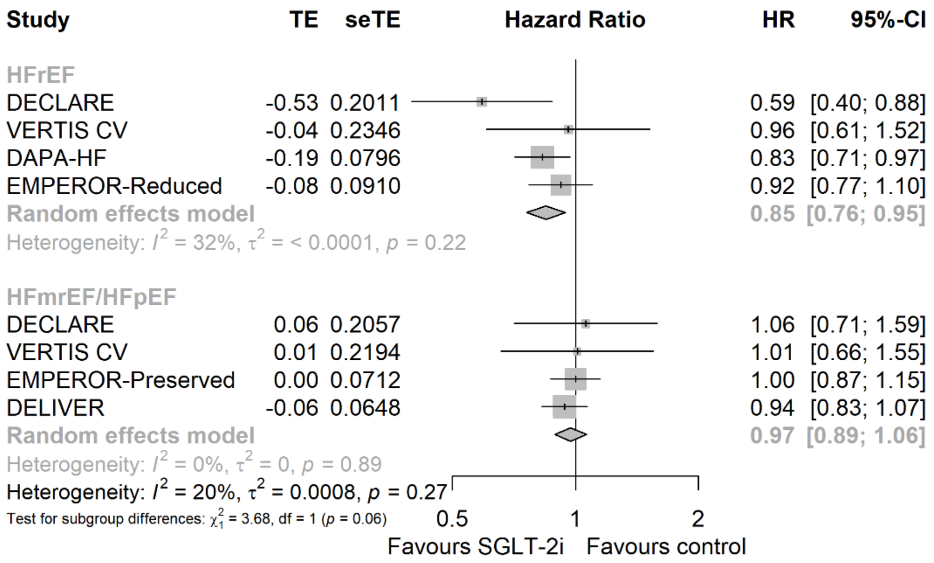

Supplemental Figure 10. The subgroup analysis of hospitalization due to heart failure according to the type of heart failure.

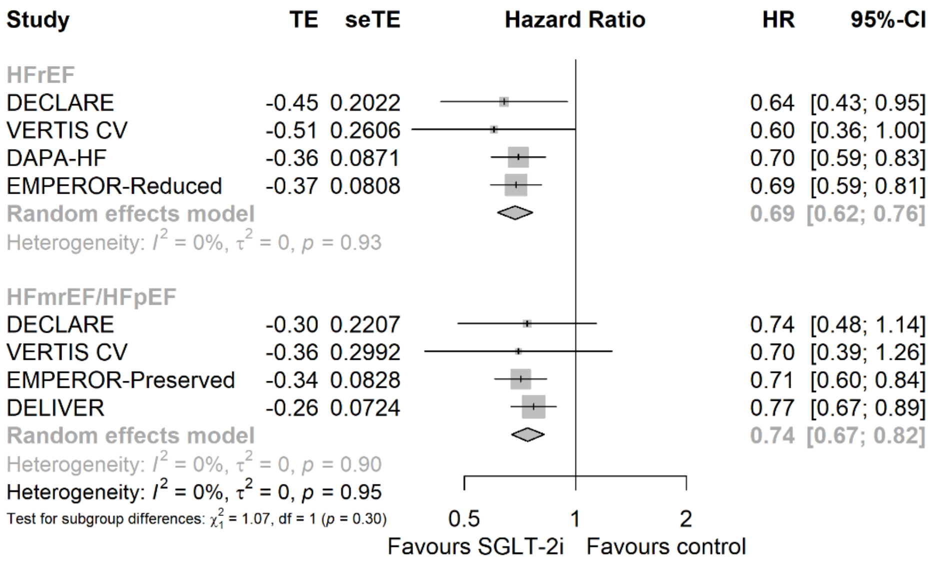

Supplemental Figure 11. The subgroup analysis of primary outcome according to the age.

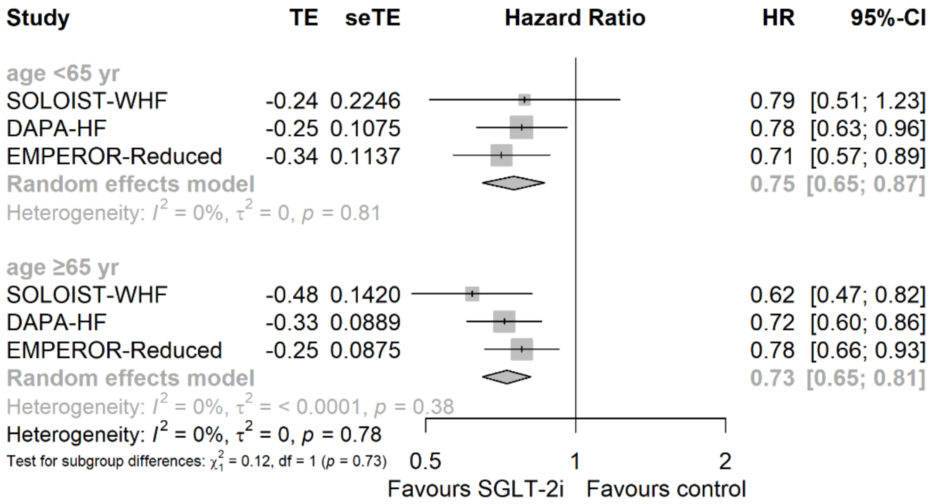

Supplemental Figure 12. The subgroup analysis of primary outcome according to the sex.

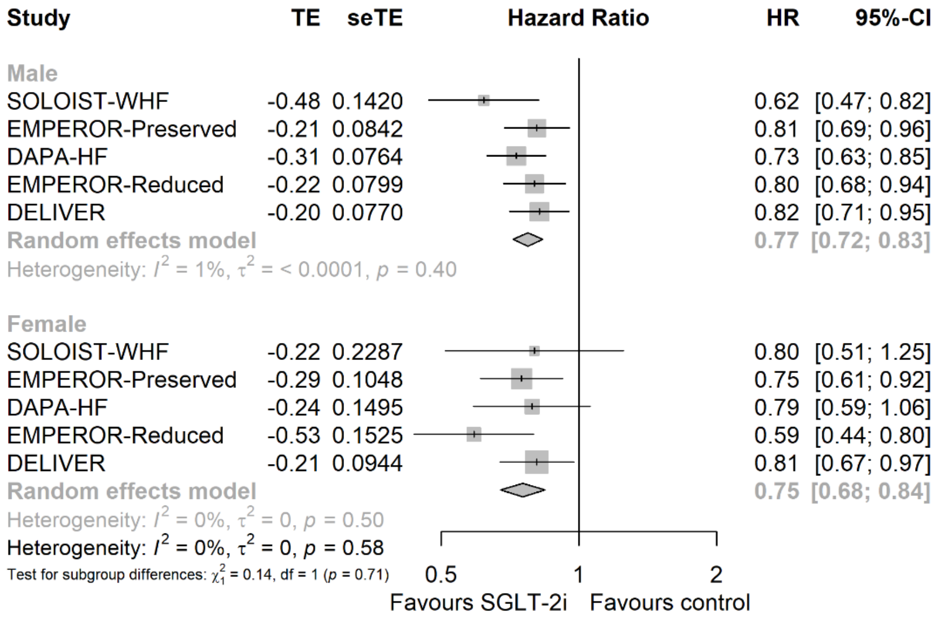

Supplemental Figure 13. The subgroup analysis of primary outcome according to the race.

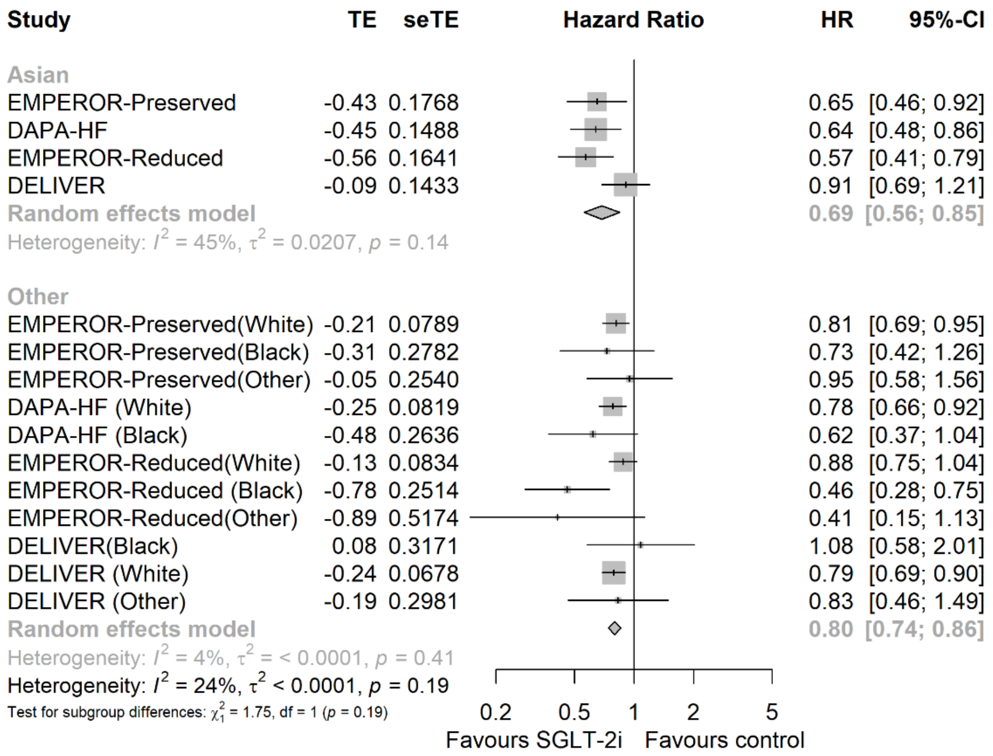

Supplemental Figure 14. The subgroup analysis of primary outcome according to the body mass index (BMI).

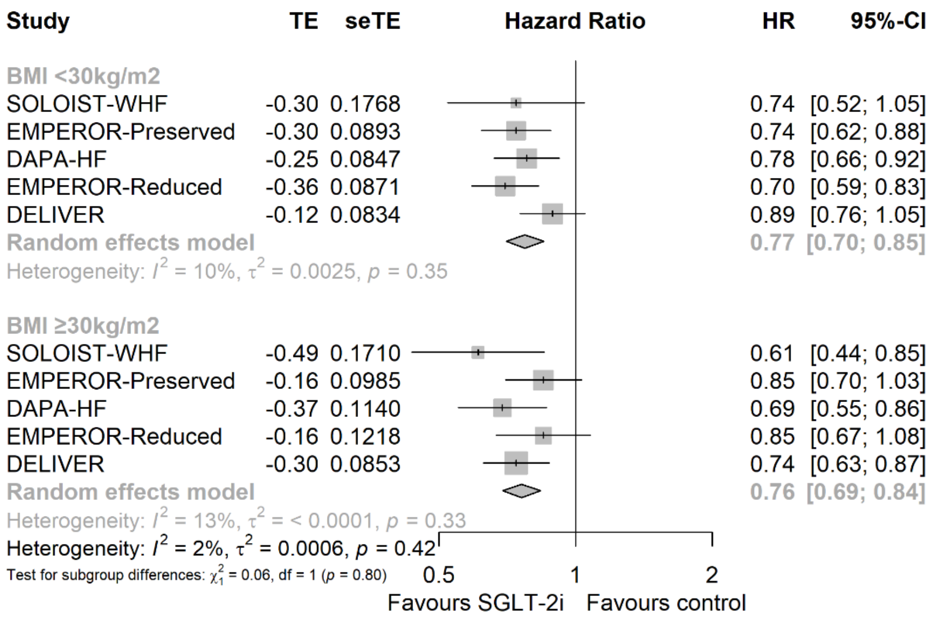

Supplemental Figure 15. The subgroup analysis of primary outcome according to the estimated glomerular filtration rate (eGFR).

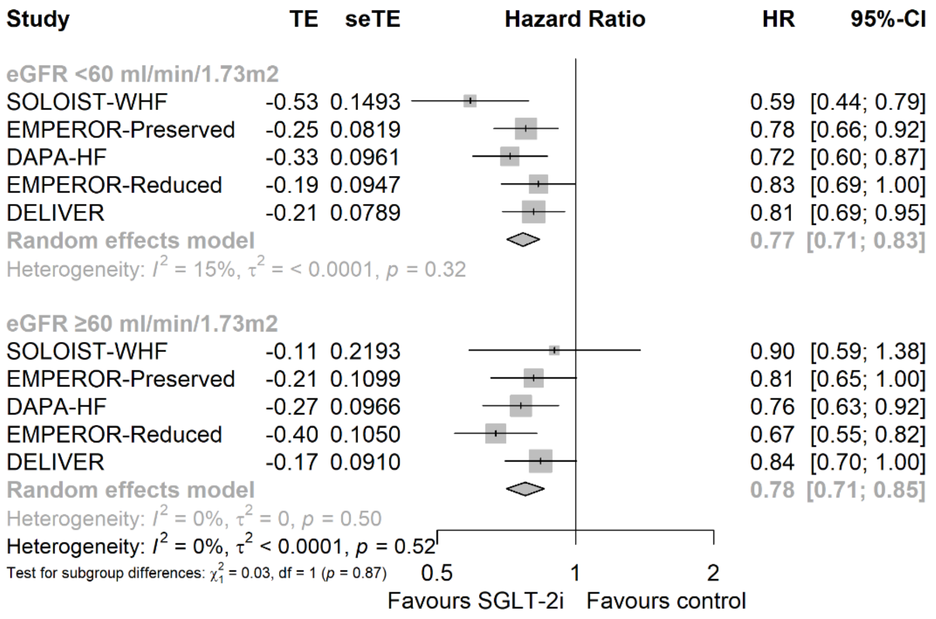

Supplemental Figure 16. The subgroup analysis of primary outcome according to the diabetes mellitus (DM).

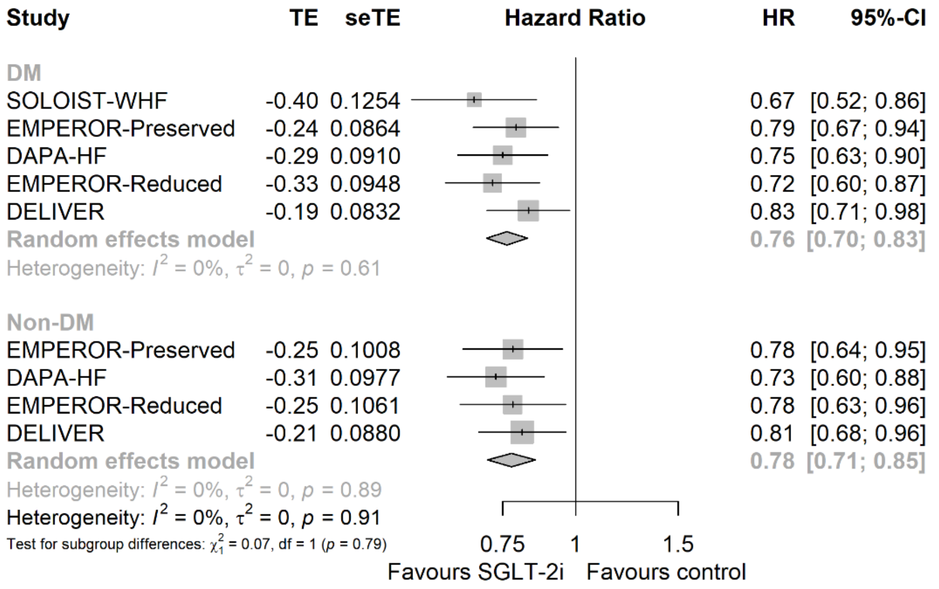

Supplemental Figure 17. The subgroup analysis of primary outcome according to the atrial fibrillation/flutter (AF).

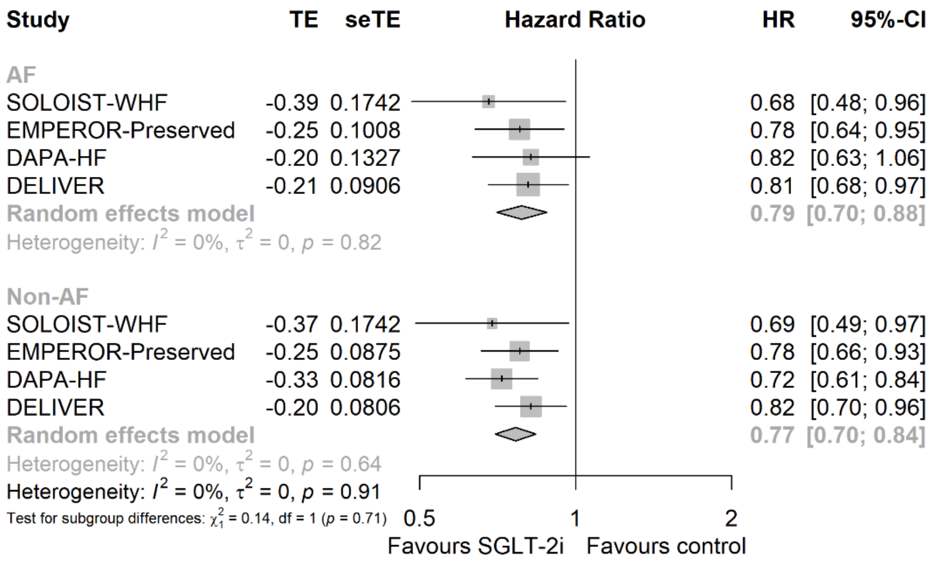

Supplemental Figure 18. The subgroup analysis of primary outcome according to the NYHA Class.

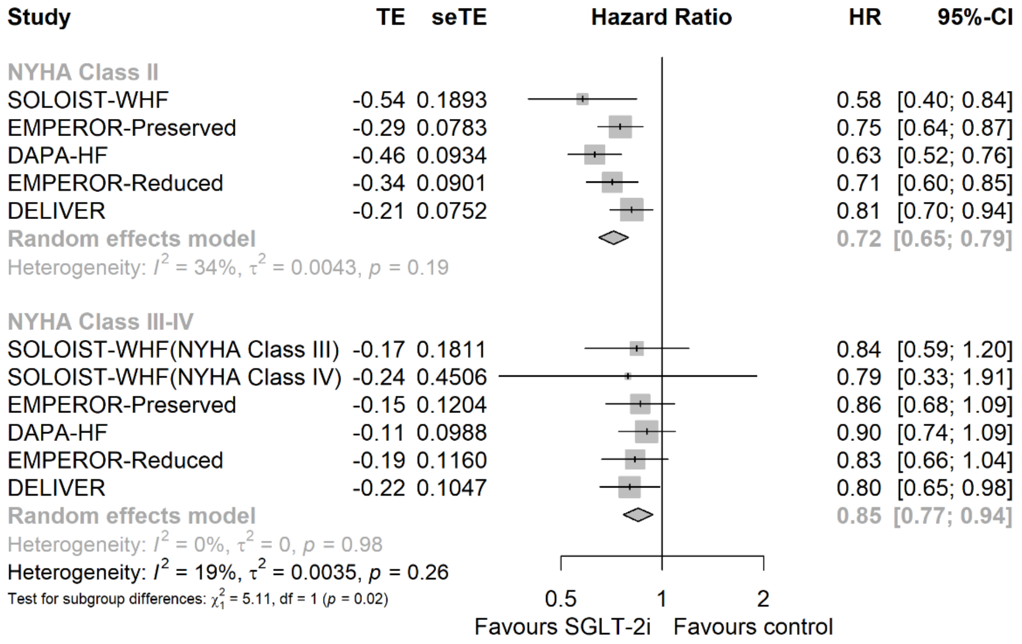

Supplemental Figure 19. The subgroup analysis of primary outcome according to the heart failure etiology.

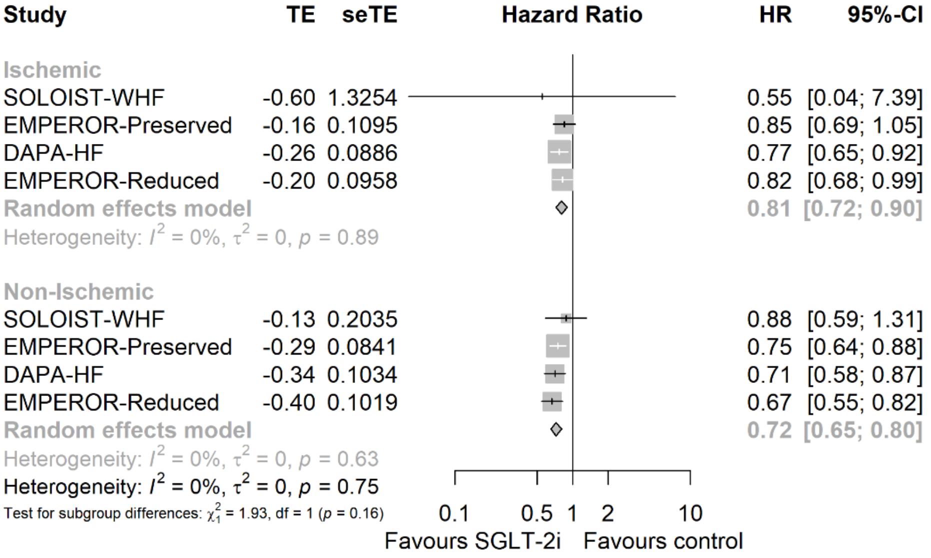

Supplemental Figure 20. The subgroup analysis of primary outcome according to the use of ARNI (angiotensin receptor-neprilysin inhibitor)/angiotensin converting enzyme inhibitor (ACEI)/ angiotensin receptor blocker (ARB).

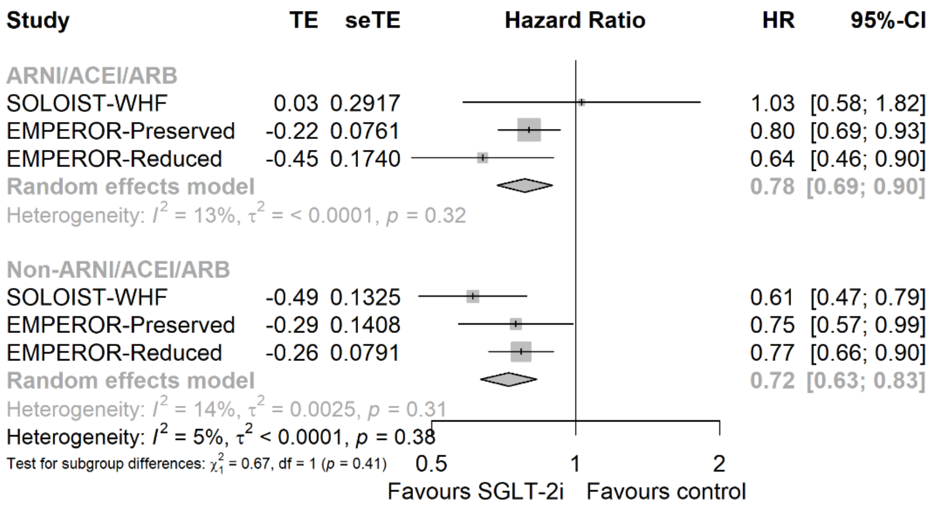

Supplemental Figure 21. The subgroup analysis of primary outcome according to the use of mineralocorticoid receptor antagonists (MRAs).

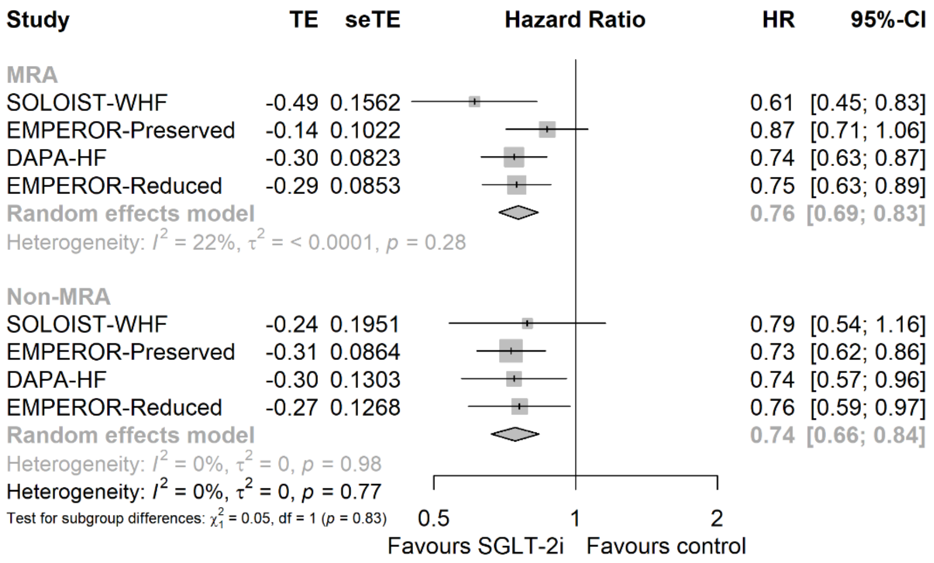

Supplemental Figure 22. The funnel plot in terms of primary outcome.

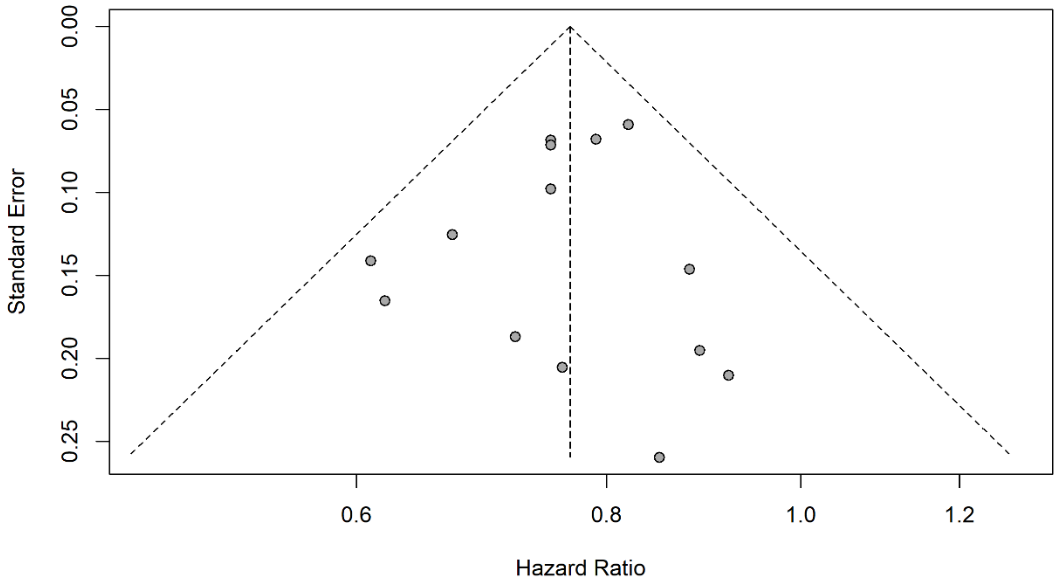

Supplemental Figure 23. The funnel plot in terms of cardiovascular death.

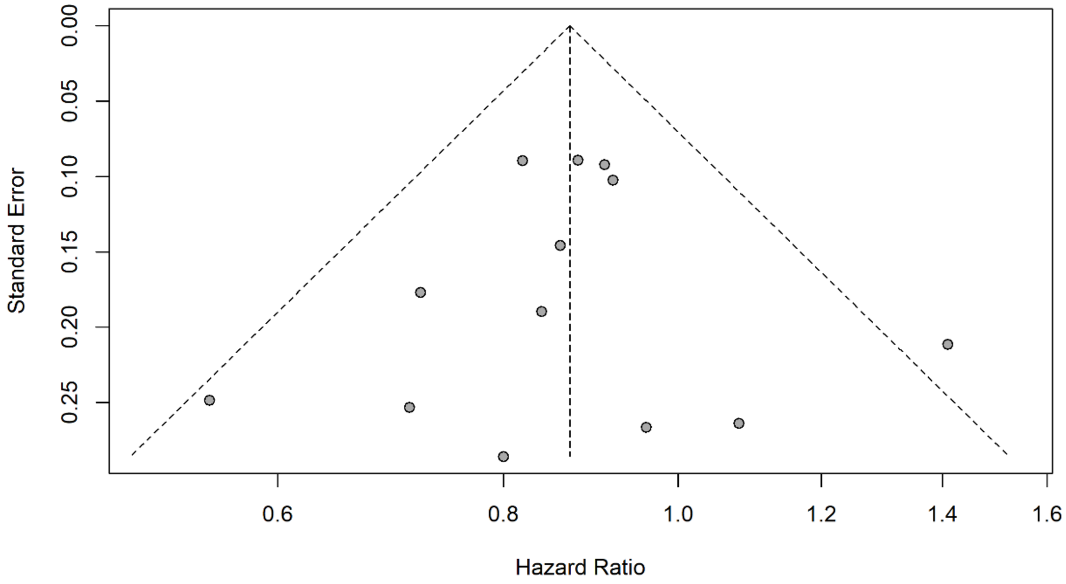

Supplemental Figure 24. The funnel plot in terms of all-cause mortality.

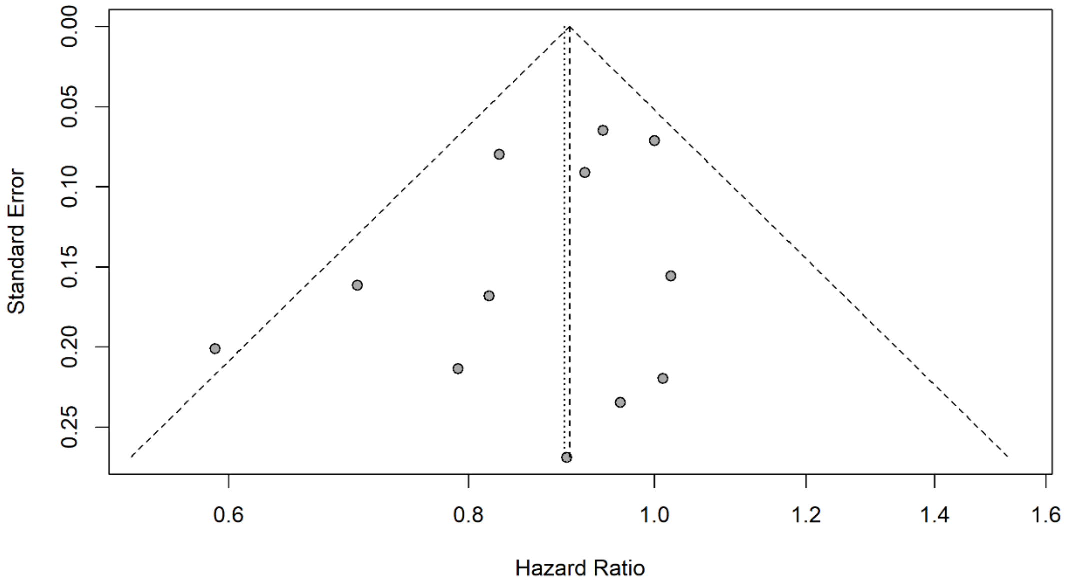

Supplemental Figure 25. The funnel plot in terms of hospitalization due to heart failure.

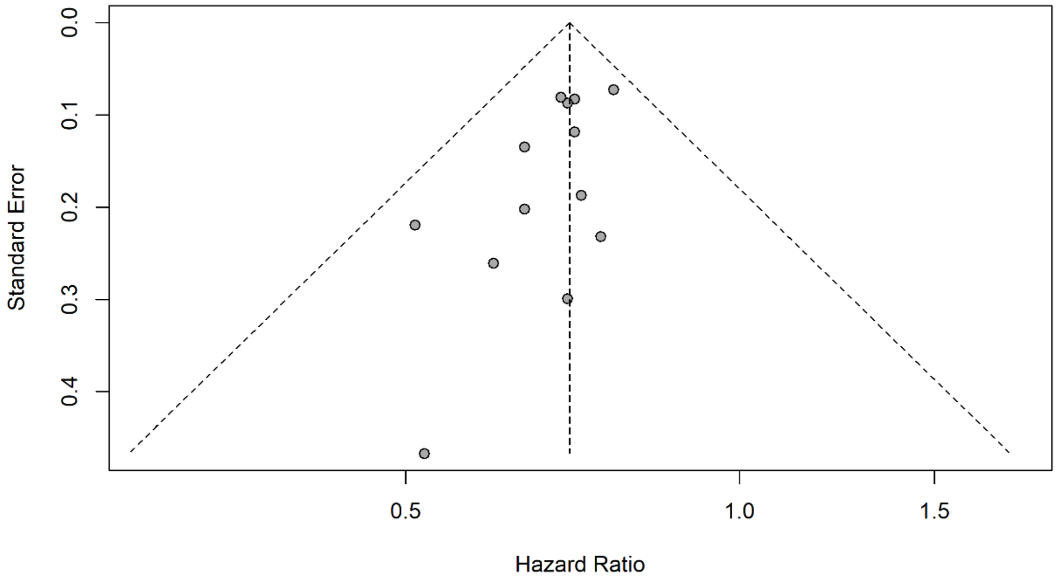

Supplemental Figure 26. The trial sequential analysis in terms of hospitalization due to heart failure.

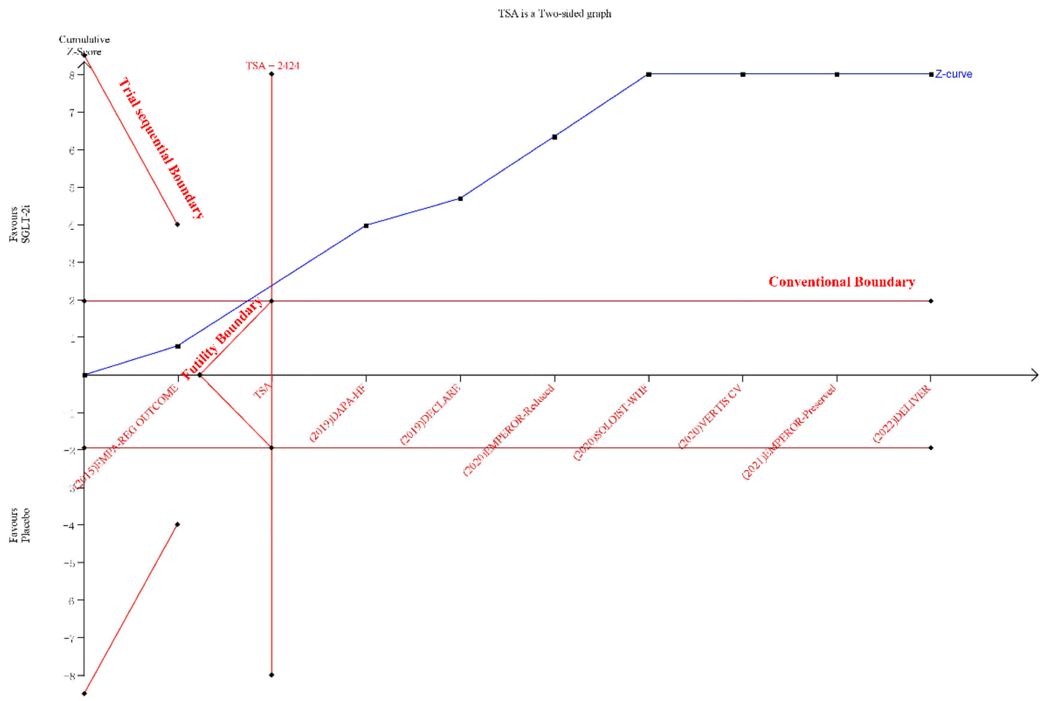

Supplement: Supplementary file 1 [file Data_Sheet_1.PDF]
